# Effects of Dietary Plant-Derived Low-Ratio Linoleic Acid/Alpha-Linolenic Acid on Blood Lipid Profiles: A Systematic Review and Meta-Analysis

**DOI:** 10.3390/foods12163005

**Published:** 2023-08-09

**Authors:** Qiong Wang, Hui Zhang, Qingzhe Jin, Xingguo Wang

**Affiliations:** State Key Lab of Food Science and Technology, Collaborative Innovation Center of Food Safety and Quality Control in Jiangsu Province, School of Food Science and Technology, Jiangnan University, Wuxi 214122, China

**Keywords:** linoleic acid/alpha-linolenic acid, blood lipid profiles, meta-analysis, randomized controlled trials

## Abstract

This meta-analysis aimed to investigate the impact of low-ratio linoleic acid/alpha-linolenic acid (LA/ALA) supplementation on the blood lipid profiles in adults. We conducted a systematic search for relevant randomized controlled trials (RCTs) assessing the effects of low-ratio LA/ALA using databases including PubMed, Embase, Cochrane, and Web of Science, as well as screened related references up until February 2023. The intervention effects were analyzed adopting weighted mean difference (WMD) and 95% confidence interval (CI). The meta-analysis indicated that low-ratio LA/ALA supplementation decreased total cholesterol (TC, WMD: −0.09 mmol/L, 95% CI: −0.17, −0.01, *p* = 0.031, I^2^ = 33.2%), low-density lipoprotein cholesterol (LDL-C, WMD: −0.08 mmol/L, 95% CI: −0.13, −0.02, *p* = 0.007, I^2^ = 0.0%), and triglycerides (TG, WMD: −0.05 mmol/L, 95% CI: −0.09, 0.00, *p* = 0.049, I^2^ = 0.0%) concentrations. There was no significant effect on high-density lipoprotein cholesterol concentration (HDL-C, WMD: −0.00 mmol/L, 95% CI: −0.02, 0.02, *p* = 0.895, I^2^ = 0.0%). Subgroup analysis showed that low-ratio LA/ALA supplementation significantly decreased plasma TC, LDL-C, and TG concentrations when the intervention period was less than 12 weeks. In the subgroup analysis, a noteworthy decrease in both TC and LDL-C levels was observed in individuals receiving low-ratio LA/ALA supplementation in the range of 1–5. These findings suggest that this specific range could potentially be effective in reducing lipid profiles. The findings of this study provide additional evidence supporting the potential role of low-ratio LA/ALA supplementation in reducing TC, LDL-C, and TG concentrations, although no significant impact on HDL-C was observed.

## 1. Introduction

Cardiovascular disease (CVD) is the leading common cause of death in the world [1]. Epidemiologic statistics show that the prevalence of CVD continues to increase, with approximately 17.9 million people dying of CVD each year, representing 32% of all deaths in the world [2,3]. This concerning trend is not only evident in industrialized nations but also rapidly increasing in emerging economies. Dyslipidemia is widely recognized as one of the major modifiable risk factors for CVD, which is characterized by elevated blood levels of total cholesterol (TC), low-density lipoprotein cholesterol (LDL-C), and triglycerides (TG), or lower levels of high-density lipoprotein cholesterol (HDL-C) [4,5]. The high incidence of dyslipidemia is related to multiple factors, including genetics, lifestyle, and environment. Improving lifestyle and diet, particularly concerning the composition of different fatty acids, has a vital effect on alleviating dyslipidemia [6].

Dietary fat, particularly n-6 and n-3 polyunsaturated fatty acids (PUFAs), is highly intertwined with lipid metabolism and overall health [7]. There is a competitive inhibitory relationship between n-6 and n-3 PUFA for desaturases, and the influence of single PUFA on certain diseases is statistically insignificant. The consumption of low-ratio n-6/n-3 PUFA has been shown to exhibit beneficial impacts on lipid metabolism and endothelial function and offers multiple benefits for preventing and treating CVD [8,9,10]. A meta-analysis conducted previously demonstrated the significant impact of a low n-6/n-3 PUFA ratio on modulating lipid profiles, with reduced serum TG level and increased serum HDL-C level [11]. Dietary n-3 PUFAs consist mainly of alpha-linolenic acid (ALA) derived from plants, as well as docosahexaenoic acid (DHA) and eicosapentaenoic acid (EPA) derived from animals. Dietary n-6 PUFAs are typified by plant-derived linoleic acid (LA). However, it is worth noting that individuals residing in inland areas often encounter limited access to seafood resources, resulting in minimal intake of rich foods in DHA and EPA. Therefore, ALA has become a major source of n-3 PUFA, which is found in different plants such as flaxseed/linseed, perilla seed, sea buckthorn seed, and walnuts. Abdelhamid showed no significant beneficial impact of ALA intake on lipid profiles in six randomized controlled trials (RCTs) [12]. A study conducted by Yue demonstrated that dietary ALA intervention improved lipid profiles by lowering TC, LDL-C, and TG concentrations [13]. Despite ALA being a precursor to long-chain PUFAs, the rate of transformation to EPA and DHA in humans remains quite modest, estimated to be approximately 3% [14]. Dietary plant and animal sources of n-3 fatty acids have different physiological roles at estimated bioequivalent intakes. The action of LA/ALA in lipid metabolism compared to n-6/n-3 long-chain PUFA has not been consistently concluded. Achieving the optimal balance of LA/ALA is crucial to sustaining physiological equilibrium influenced by genetics and surroundings. A previous randomized controlled trial showed that low-ratio LA/ALA supplementation reduced body weight, TC, and LDL-C [15,16]. Several research findings have highlighted the potential link between a higher LA/ALA ratio, increased obesity risk, and heightened inflammatory factors [17]. However, some results are just the opposite.

Previous studies have conducted meta-analyses to examine the correlation between lipid and dietary intake of ALA. However, limited research has been conducted on the influence of the LA/ALA ratio, and the findings have sparked considerable controversy. Performing a review of the literature may clarify the relationship between dietary intake low-ratio LA/ALA and blood lipids. Thus, this meta-analysis sought to investigate the influence of low-ratio LA/ALA on TG, TC, LDL-C, and HDL-C concentrations.

## 2. Materials and Methods

### 2.1. Search Strategy and Selection Studies

The present investigation complied with the guidelines summarized in the Preferred Reporting Items for Systematic Reviews and Meta-Analyses (PRISMA) checklist [18]. The research methodology has been registered on the PROSPERO website (CRD42020216635). To retrieve relevant data, an extensive search was performed across multiple databases, including Web of Science, Embase, Cochrane, and PubMed, up until February 2023. The text terms included: (“alpha-linolenic acid” OR “α-linolenic acid” OR “linoleate” OR “linoleic acid” OR “linoleate” OR “linoleic acid/alpha-linolenic acid” OR “linoleic acid/a-linolenic acid” OR “LA/ALA”) AND (“blood lipid” OR “cholesterol” OR “TC” OR “low-density lipoprotein cholesterol” OR “LDL-C” OR “high-density lipoprotein cholesterol” OR “HDL-C” OR “triglyceride” OR “TG”). Additionally, we manually searched bibliographies of the included studies and related reviews to determine any additional studies that may be pertinent to our investigation.

### 2.2. Inclusion Criteria

The inclusion criteria for relevant articles are listed below: (1) the investigation solely encompassed RCTs conducted on human subjects in either parallel or crossover design; (2) the study examined the influence of LA/ALA ratio on TG, TC, HDL-C, and LDL-C; (3) the study population consisted of adult participants 18 years of age and older, with the exception of pregnant women; (4) the test group, which was supplied with LA and ALA by diet or supplementation, differed from the control group only in the proportion of fatty acids; (5) intervention type includes oral feeding, excluding enteral nutrition and intravenous inputs; (6) the article explicitly reported the LA/ALA ratio, or the ratio could be derived using appropriate calculations; (7) provide sufficient information to extract or calculate values and standard deviations (SD) of changes in blood lipid; (8) the duration of intervention was at least 2 weeks.

Studies meeting the following criteria were excluded: (1) using ALA supplementations together with other functional oils and fatty acids, including fish oil, DHA, DPA, EPA, and conjugated linoleic acid (CLA); (2) only ALA or LA content, not LA/ALA ratio; (3) parenteral nutrition, animal, or in vitro studies; (4) those that did not report the baseline or end values of outcome variables; (5) non-original research, duplicate articles and non-English articles.

### 2.3. Data Extraction

Two reviewers (WQ and JQ) independently assessed the methodological quality of the included RCTs and extracted relevant data of the eligible trials using the Cochrane Handbook and a standard Excel, respectively. Any controversy or disagreement among study selection was reconciled with the third (LR). The relevant data was abstracted from eligible articles: the first author’s name and publication year, participant demographics, health status, sample size, country of origin, LA/ALA ratio, duration, and the mean (SD) changes of blood lipid at baseline and endpoint. When the outcome was published multiple times in different time points and trials, data from the trials with the largest and longest duration of the intervention was extracted.

### 2.4. Statistical Analysis

All statistical analyses were conducted using STATA 14.0 software. To determine the effect sizes of the average changes in lipid concentrations, we extracted the mean changes and standard deviations (SD) from the included studies. In cases where SDs were not directly reported, conversion formulas were employed to calculate them [19]. Additionally, for data presented solely in graphical form, we utilized the GetData Graph Digitizer software to digitally extract and quantify the relevant information. All lipid levels were collated as mmol/L; mg/dL reported lipid values were converted to mmol/L by multiplying by 0.0113 (for triglycerides) and 0.0259 (for cholesterol). The statistical significance of net changes was indicated by weighted mean difference (WMD) with 95% CI. To assess the heterogeneity among the included studies, we employed statistical measures such as the *p* value and I^2^ index. A *p*-value below 0.05 was considered statistically significant. The degree of heterogeneity was categorized as low (I^2^ ≤ 25%), moderate (25% < I^2^ ≤ 50%), or high (50% < I^2^ ≤ 75%), respectively.

Additionally, predetermined subgroup analyses were performed to determine the relationship between various factors and heterogeneity, including age (≤45 and >45 years), regions and countries, healthy status, supplementation duration (<12 and ≥12 weeks), and LA/ALA ratio. Meta-regression with restricted maximum likelihood (REML) was carried out to estimate the association between the effect size of blood lipids and supplementation duration. We conducted internal sensitivity analyses by the omission of one trial in each round to detect the influence of one study on the validity of the overall effect sizes. The possible publication bias was evaluated with Begg’s and Egger’s tests between included trials.

## 3. Results

### 3.1. Description of Studies

**Figure 1:**
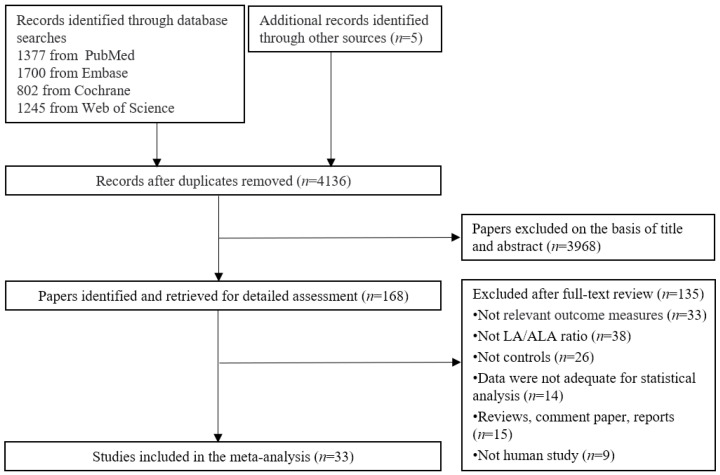
Screening flowchart of this study.

The screening flowchart is illustrated in Figure 1. A total of 5129 publications were initially retrieved from four databases; of them, 993 duplicate records were removed. Based on screening titles and abstracts, we included 168 articles for further examination of the full text. Finally, a total of 33 trials were eligible for the current meta-analysis.

The detailed information of 33 papers is listed in Table 1 and Table S1. The studies included 2204 participants with sample sizes ranging from 11 to 243 participants. Of a total of 33 studies, 12 studies were conducted in healthy subjects [20,21,22,23,24,25,26,27,28,29,30,31], 11 studies in subjects with dyslipidemia [15,32,33,34,35,36,37,38,39,40,41], four studies in subjects with type 2 diabetes mellitus [42,43,44,45], two studies in subjects with overweight or obese [46,47], two studies in subjects with metabolic syndrome [48,49], one study in subjects with cardiovascular [50], and one study in subjects with non-alcoholic fatty liver [51]. Twenty-two articles were performed using parallel, and eleven articles had crossover designs. The mean age of the included trials was between 24.5 [29] and 64 years [36], and the BMI ranged from 21.9 [26] to 34.5 [42] approximately. Selected trials were performed in North America (*n* = 11), Europe (*n* = 14), Asia (*n* = 6), and Oceania (*n* = 2). Non-smoking participants were included in 15 trials, while the subjects of mixed smokers were attended in 11 studies. Two studies included only female subjects [34,36], and seven trials included only male subjects [21,25,27,29,36,37,38]. The LA/ALA ratio varies between 0.14 [48] and 228.2 [51] for an intervention duration of 3–104 weeks in the studies.

The total energy intake of subjects with different LA/ALA ratios was constant throughout the study. In all but three studies [40,43,45], the macronutrients (protein, carbohydrate, and fat) as a percentage of total daily energy were the same between the low-ratio LA/ALA group and the control group. Out of a total of 33 studies, 7 studies had significant differences in PUFA [21,23,34,43,45,48,49], 3 studies had significant differences in MUFA and PUFA [26,28,31], 1 studies had significant differences in SFA and PUFA [40], and 2 study had significant differences in SFA, MUFA, and PUFA [15,39]. In the high LA/ALA ratio group, the dietary intake range of LA was between 3.3% (6.2 g) of total energy/d [43] and 16.22% of total energy/d [46], and the dietary intake range of ALA was between 0.07% of total energy/d [46] and 1.6% of total energy/d [25] approximately. In the low LA/ALA ratio group, the dietary intake range of LA was between 1.49% (3.1 g) of total energy/d [33] and 13.26% (26.0 g) of total energy/d [43], and the dietary intake range of ALA was between 0.7% of total energy/d [28] and 7.51% of total energy/d [46] approximately. The LA/ALA ratio can be achieved by blending one or multiple oils. The LA/ALA ratio was increased via the supplementation of sunflower oil, safflower oil, corn oil, canola oil, olive oil, and soybean oil. The LA/ALA ratio was decreased via the supplementation of flaxseed/linseed oil, rapeseed oil, camelina oil, echium oil, hempseed oil, walnut oil, canola oil, and olive oil.

### 3.2. Quality Assessment

The quality assessment of the included studies was evaluated in Table 2. A total of 15 trials provided sufficient data regarding the methods used for random sequence generation, while in 18 trials, the methods employed for random sequence generation were unknown. Only seven provided clear information on allocation concealment, indicating a low risk of bias. The risk of bias for the remaining studies in this regard is unclear. A double-blind design was performed on 19 assessed trials, and a single-blind design was conducted on five assessed trials. In addition, a considerable number of studies exhibit a lack of information regarding the blinding of outcome assessment, with six trials observing a low risk of bias. The risk of bias was unclear for six trials due to incomplete outcome data. Finally, six studies were categorized as being at low risk of selective outcome reporting since they provided a study protocol. None of the studies exhibited other sources of bias.

### 3.3. Effect of Low-Ratio Linoleic Acid/Alpha-Linolenic Acid on Blood Lipid Profiles

**Figure 2:**
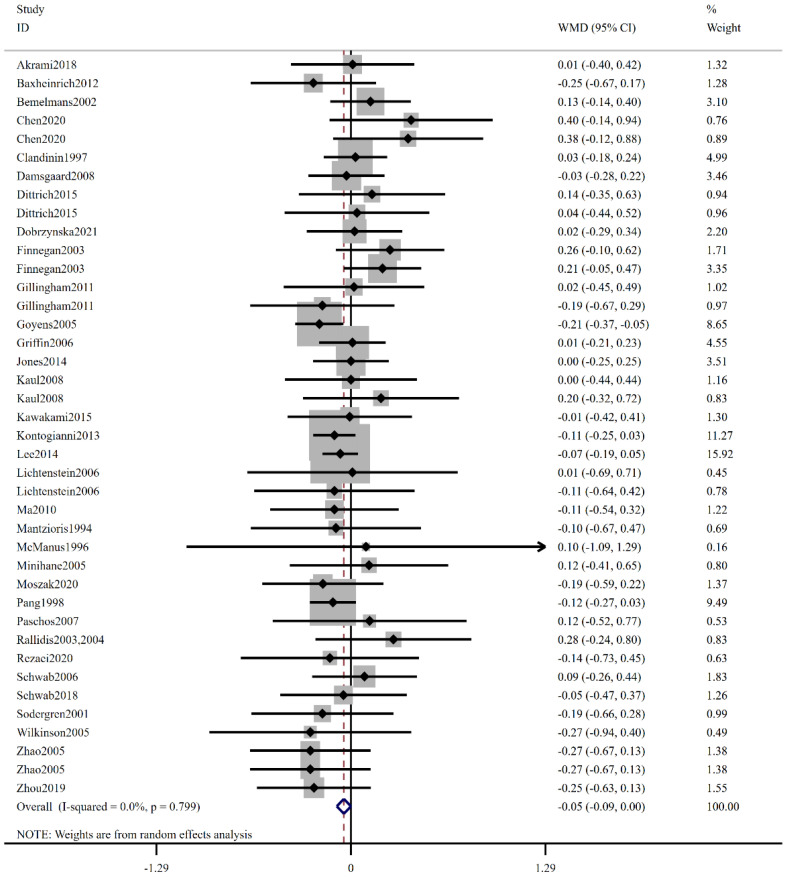
The effect of low-ratio LA/ALA on TG. Refs. [15,20,21,22,23,24,25,26,27,28,29,30,31,32,33,34,35,36,37,38,39,40,41,42,43,44,45,46,47,48,49,50,51].

Figure 2 illustrates the forest plot depicting the impact of low-ratio LA/ALA intake on plasma TG. A total of thirty-three studies provided data on the impact of LA/ALA on TG level. The pooled analysis revealed that the low ratio of LA/ALA intake significantly decreased plasma TG (WMD: −0.05 mmol/L, 95% CI: −0.09, 0.00, *p* = 0.049). No significant heterogeneity was observed among the studies (I^2^ = 0.0%, *p* = 0.799). Subgroup analysis, as presented in Table 3, was conducted based on low-ratio LA/ALA, regions, health status, age, BMI, smoking, and duration. Stratification by health status demonstrated a noteworthy decrease in plasma TG levels among healthy subjects (WMD: −0.08 mmol/L, 95% CI: −0.15, −0.01, I^2^ = 0.0%, *p* = 0.022). Low-ratio LA/ALA intake significantly decreased TG level for people with age ≤ 45 (WMD: −0.09 mmol/L, 95% CI: −0.15, −0.03, I^2^ = 0.0%, *p* = 0.004). Subgroup analyses based on intervention duration indicated a statistically significant reduction in plasma TG with low-ratio LA/ALA supplementation when the intervention duration < 12 weeks (WMD: −0.09 mmol/L, 95% CI: −0.15, −0.04, I^2^ = 0.0%, *p* = 0.001).

**Figure 3:**
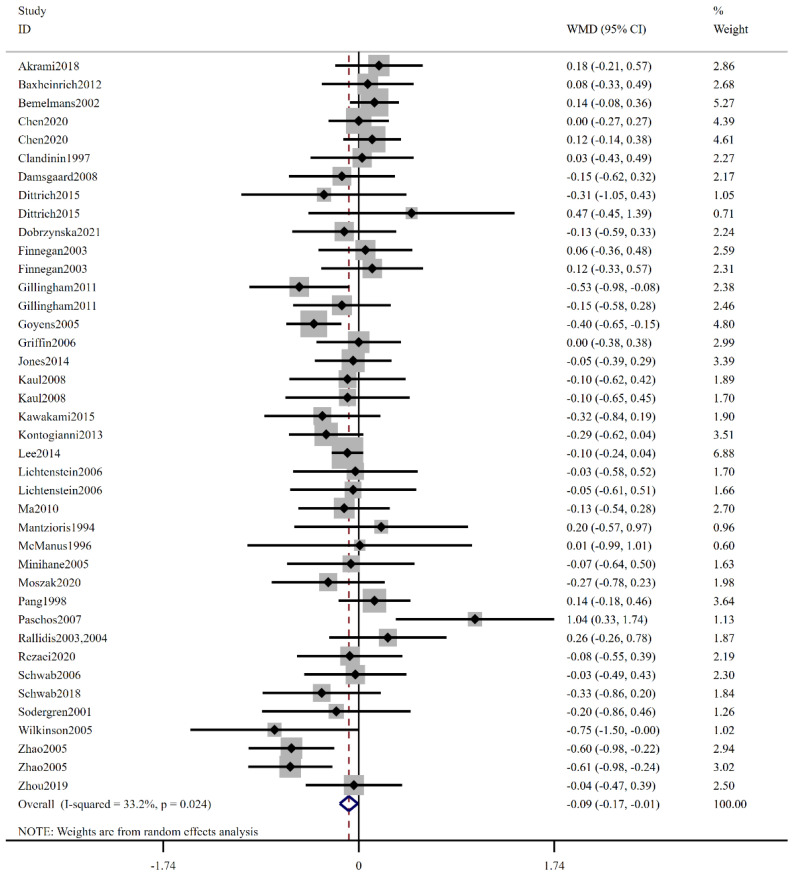
The effect of low-ratio LA/ALA on TC. Refs. [15,20,21,22,23,24,25,26,27,28,29,30,31,32,33,34,35,36,37,38,39,40,41,42,43,44,45,46,47,48,49,50,51].

As shown in Figure 3, the pooled analysis, encompassing 40 comparisons from 33 studies, identified that the low-ratio LA/ALA group had obvious effects on the plasma cholesterol level compared to the high-ratio LA/ALA group (WMD: −0.09 mmol/L, 95% CI: −0.17, −0.01, *p* = 0.031). The included trials demonstrated substantial statistical heterogeneity as determined using the I^2^ test (I^2^ = 33.2%, *p* = 0.024). To further explore this heterogeneity, a stratified analysis was conducted based on different levels of low-ratio LA/ALA (≤1, 1–5, and ≥5), as outlined in Table 3. The results showed that the difference between the control and experimental groups was significant when the low-ratio LA/ALA was within the range of 1–5 (WMD: −0.12 mmol/L, 95% CI: −0.23, −0.01, *p* = 0.031). Moreover, our findings indicated that low-ratio LA/ALA intake in North America had a significant decreasing influence on TC levels (WMD: −0.21 mmol/L, 95% CI: −0.33, −0.07, *p* = 0.002). Subgroup analysis suggested the effectiveness of low-ratio LA/ALA supplement on TC level in healthy subjects (WMD: −0.15 mmol/L, 95% CI: −0.28, −0.03, *p* = 0.016). When the trials were subgrouped by age, BMI, and duration, the significant impact of low-ratio LA/ALA on TC was evidenced in the groups of age ≤ 45, BMI ≥ 30, and intervention period <12 weeks.

**Figure 4:**
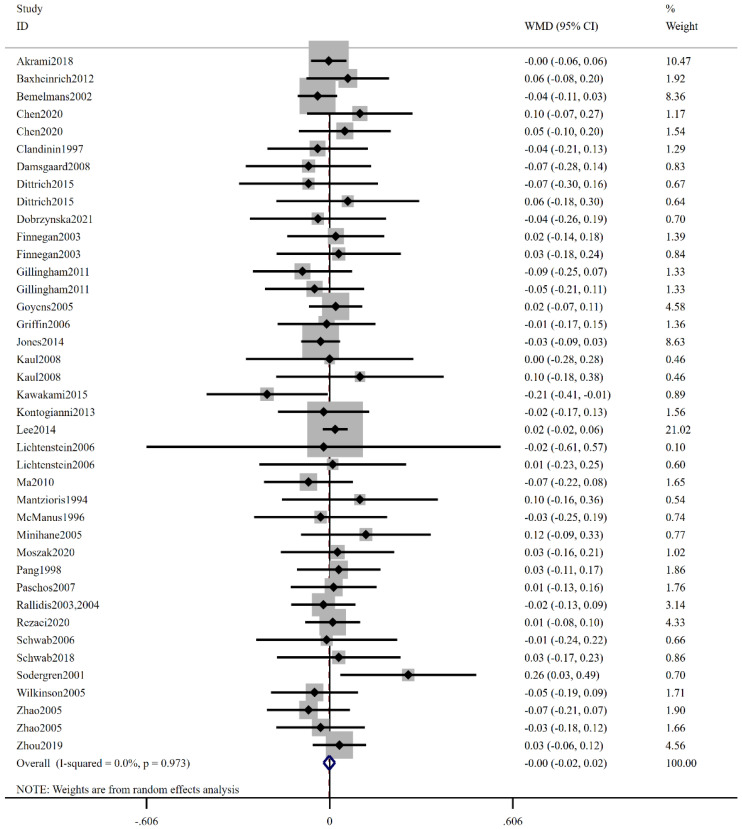
The effect of low-ratio LA/ALA on HDL-C. Refs. [15,20,21,22,23,24,25,26,27,28,29,30,31,32,33,34,35,36,37,38,39,40,41,42,43,44,45,46,47,48,49,50,51].

As shown in Figure 4, overall results revealed that low-ratio LA/ALA consumption did not significantly influence blood HDL-C compared to high-ratio LA/ALA (WMD: −0.00 mmol/L, 95% CI: −0.02, 0.02, *p* = 0.895), with a low level of heterogeneity (I^2^ = 0.0%, *p* = 0.973). Upon subgroup analysis based on participant characteristics such as region, duration, low-ratio LA/ALA intake, age, BMI, smoking, and health status, no significant variation in the influence of low-ratio LA/ALA on HDL-C level was observed across all subgroups (Table 3).

**Figure 5:**
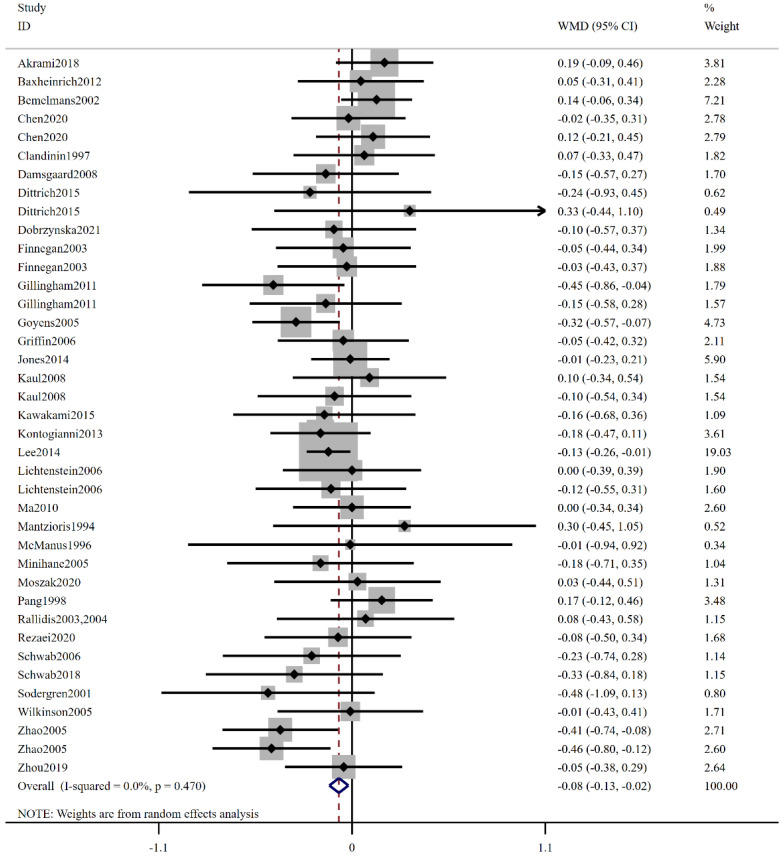
The effect of low-ratio LA/ALA on LDL-C. Ref [15,20,21,22,23,24,25,26,27,28,29,30,31,32,33,34,35,36,38,39,40,41,42,43,44,45,46,47,48,49,50,51].

In addition, 32 trials with 39 comparisons showed that low-ratio LA/ALA supplementation significantly reduced LDL-C level (WMD: −0.08 mmol/L, 95% CI: −0.13, −0.02, *p* = 0.007) (Figure 5). The included trials were not statistically heterogeneous as determined using the I^2^ test (I^2^ = 0.0%, *p* = 0.470). The results presented in Table 3 indicated a statistically significant difference when the intake of low-ratio LA/ALA fell within the range of 1–5 (WMD: −0.10 mmol/L, 95% CI: −0.18, −0.02, *p* = 0.011). When the trials were categorized by region, the combined analysis revealed a decrease in plasma LDL-C concentration, specifically in North America (WMD: −0.14 mmol/L, 95% CI: −0.23, −0.05, *p* = 0.003). Furthermore, categorized by health status, the pooled effect analysis demonstrated a reduction in LDL-C concentration among subjects with dyslipidemia (WMD: −0.14 mmol/L, 95% CI: −0.25, −0.03, *p* = 0.007), or T2DM (WMD: −0.13 mmol/L, 95% CI: −0.24, −0.01, *p* = 0.007). LDL-C levels were significantly lower in subgroup analyses of subjects with a BMI between 25–30 (WMD: −0.11 mmol/L, 95% CI: −0.20, −0.02, *p* = 0.014) and BMI ≥ 30 (WMD: −0.11 mmol/L, 95% CI: −0.21, −0.01, *p* = 0.031). Regarding the stratification based on duration, the extracted results indicated a significant distinction between the experimental group consuming low-ratio LA/ALA and the control group consuming high-ratio LA/ALA, specifically when the duration of intervention was less than 12 weeks.

### 3.4. Publication Bias and Sensitivity Analysis

Visual examination of Egger’s and Begg’s regression test revealed potential publication bias in the meta-analyses (Figure S1). Begg’s funnel plots indicated no publication bias for TG (*p* = 0.351) TC (*p* = 0.650). HDL-C (*p* = 0.421), and LDL-C (*p* = 0.304). Egger’s funnel plots indicated no publication bias for TG (*p* = 0.070), TC (*p* = 0.925), HDL (*p* = 0.847), or LDL (*p* = 0.984).

Sensitivity analysis was conducted using iteratively excluding individual studies, and the results showed that none of the studies had a substantial impact on the pooled effect of TG (Figure S2), TC (Figure S3), HDL-C (Figure S4), and LDL-C (Figure S5).

## 4. Discussion

Dyslipidemia is linked to an increased likelihood of CVD, type-2 diabetes, atherosclerosis, and various diseases related to obesity [4]. Several fatty acids used in food for special medical purposes have been exploited to improve blood lipids and prevent CVD and atherosclerosis, such as DHA [52], EPA [53], and medium-chain fatty acids (MCFA) [54]. The plant-derived ALA is one of the main fatty acid components of flaxseed/flaxseed oil, perilla oil, sea buckthorn seed oil, and walnut oil. The previous studies demonstrated that low-ratio LA/ALA effectively decreased TC, TG, and LDL-C levels, as well as increased HDL-C levels [55,56,57,58]. In addition, several trials also reported that low-ratio LA/ALA did not show to affect lipid profiles [59,60]. Thus, the aim of this meta-analysis was to comprehensively evaluate the effectiveness of low-ratio LA/ALA supplementation in improving blood lipid profiles.

Prior analyses have primarily emphasized the impact of ALA and animal-derived n-6/n-3 PUFA on lipid profiles. This is the first meta-analysis evaluating the influence of low-ratio LA/ALA on blood lipids. This result revealed that dietary low-ratio LA/ALA significantly lowered TG, TC, and LDL-C levels compared to high-ratio LA/ALA controls. Nevertheless, low-ratio LA/ALA supplementation did not significantly increase the blood HDL-C levels. Although the specific mechanism of low-ratio LA/ALA on plasma lipids has not been demonstrated, several possible mechanisms have been reported. The liver, being a vital organ, assumes a pivotal role in the intricate process of synthesizing fatty acids and TG. Low-ratio LA/ALA downregulated key enzymes that play a role in the synthesis of fatty acid and cholesterol within the liver, thus improving lipid metabolism disorders [38]. The decrease in plasma TC via low-ratio LA/ALA intake was attributed to the reduction in both free TC and esterified TC. ALA-rich diet increases cholesterol synthesis and turnover via transferring more TC into the bile [61]. Low-ratio LA/ALA reduces TG accumulation via simultaneously stimulating β-oxidation and inhibiting lipid biosynthesis pathways (coenzyme A carboxylase, fatty acid synthase, diacylglycerol acetyltransferase, etc.) [62]. Low-ratio LA/ALA significantly inhibits the TG and TC biosynthesis pathway by suppressing the mRNA expression of key regulatory proteins such as sterol regulatory element-binding protein [63]. Furthermore, low-ratio LA/ALA can inhibit the production of a range of inflammatory proteins involving the regulation of peroxisome proliferator-activated receptors (PPAR) and the suppression of nuclear factor-kappa B (NF-κB) [64,65]. Therefore, by implementing low-ratio LA/ALA in dietary interventions, there is a potential enhancement in lipid metabolism and a decrease in the likelihood of developing CVD and other obesity-related health problems.

Some studies have reported that plasma TG, TC, and LDL-C are reduced [40] or remain unchanged after supplementation with low-ratio LA/ALA [23,35,46]. According to the results of the study, there was some heterogeneity in the variation of lipid profiles. Subgroup analyses by geographic region showed a significant downward trend in TG, TC, and LDL-C in North America and Europe. The effects of low-ratio LA/ALA on TC and LDL-C levels were more pronounced in North America than in other regions, such as Europe and Asia. Regional disparities exist in dietary habits, encompassing not only variations in nutritional content but also differences in dietary patterns that impact alterations in blood lipid profiles. The reason for this result may be that ethnicity and genotype from different regions may be different. This hypothesis needs to be evaluated with additional data from clinical trials of genotype differences.

Furthermore, the impact of low-ratio LA/ALA supplementation on TG and TC was significant only in a subgroup of healthy subjects, and the effect of low-ratio LA/ALA intake on LDL-C was notable in the subgroups of subjects with dyslipidemia and diabetes. This may be due to the taking of drugs in hyperlipidemic subjects, the impact of inflammatory factors associated with dyslipidemia, and pathological causes of other diseases. Consumption of ALA-rich diets can improve dyslipidemia by inhibiting hepatic lipogenesis and reducing various metabolic pathways of fat [42,66]. In a cross-sectional study, increasing ALA intake by 1 g per day was associated with a 5% reduction in mortality from dyslipidemia-associated CVD [67]. Several pieces of evidence suggest that the consumption of ALA exerts significant positive effects on metabolic syndrome and T2DM, particularly in improving TG and reducing platelet aggregation [68]. The presence of a high proportion of ALA in serum phospholipids in primary dyslipidemia was negatively correlated with the burden of plaque in the carotid and femoral arteries [69]. Considering the direct effects of ALA on gut-derived lipids, ALA may induce complex compensatory interactions between the human liver and the gut. Hence, to further elucidate the impact of ALA on LDL-C concentrations, future intervention studies should focus on conducting precise RCTs.

For both TC and LDL-C, the lowering is more evident with increasing BMI. The impact of low ratio LA/ALA on total cholesterol was more pronounced in young subjects with BMI ≤ 25. This phenomenon may be explained using different mechanisms for triglyceride and cholesterol [70,71,72]. The studies grouped by age categories indicated that low-ratio LA/ALA intake reduced TG and TC only in the under 45 years of age subgroup. Older adults were more likely to present elevated dyslipidemia and inflammation, as well as increased expression of relevant enzymes. The interaction between LA and ALA is complex, and high levels of LA can counteract the anti-inflammatory effects of ALA [73]. LA and ALA compete for the same biosynthetic enzymes and elicit distinct physiologic actions in the human body. Subgroups with lower LA/ALA ratios (1 to 5) exhibited more pronounced reductions in TC and LDL-C than subgroups with ratios above or below this range. When the range of low-ratio LA/ALA fell below 1 or exceeded 5, it could not exert a significant health effect. These findings indicated that maintaining a low LA/ALA ratio within this specific range may represent an optimal approach for reducing specific lipid markers.

The reduction in TG, TC, and LDL-C was most pronounced during a short period (<12 weeks) of supplementation with a low LA/ALA ratio. However, when the intervention duration was ≥12 weeks, TG, TC, and LDL-C had an upward trend, but it was not significant. According to Abdelhamid (2018), they found that the beneficial effects of ALA were more pronounced during the 1–2 years intervention than beyond 2 years [12]. Our findings show a similar trend that short-term supplementation with a low ratio of LA/ALA may exhibit more desirable lipid profiles. The decreased health effects of low-ratio LA/ALA supplementation may be due to poor compliance among participants. With the passage of time, there was a noticeable deviation in dietary composition from the prescribed regimen, leading to changes in the LA/ALA ratio in daily intake. The impact of a low LA/ALA ratio on lipid-related indices diminished. Therefore, future research is needed to conduct long-term and highly compliant intervention studies in patients with hyperlipidemia to better evaluate the mechanism and effect of low-ratio LA/ALA.

The present study systematically revealed the relationship between plant-derived low-ratio LA/ALA and blood lipids for the first time. The low-ratio LA/ALA significantly decreased plasma TC, LDL-C, and TG concentrations. Our study was characterized by strong statistical power, as it encompassed 33 RCTs and a sample size of 2204 subjects. However, it is important to acknowledge that our meta-analysis also possesses certain limitations and drawbacks. The impact of low-ratio LA/ALA on blood lipid profiles may be confounded by additional fatty acids and bioactive constituents found in vegetable oils. Some research that provided incomplete information and inaccurately divided into groups has the potential to affect the results of subgroup analysis. It is also worth noting that the influence of additional underlying elements, like variances in genetics and alterations in lifestyle, remained challenging to adequately evaluate. The small sample sizes of each study and the use of a crossover design in some studies to increase the sample size may have some influence on the results of the argumentation.

## 5. Conclusions

The investigation into the impact of the LA/ALA ratio on blood lipids holds practical importance in treating and managing dyslipidemia and CVD. This meta-analysis suggested that low-ratio LA/ALA supplementation significantly decreased plasma TG, TC, and LDL-C concentrations. Dietary LA/ALA ratio had no influence on plasma HDL-C concentrations. LA/ALA ranging from 1 to 5 had a more potent reducing impact on TC and LDL-C compared to the ratio above or below this range. Reliable evidence shows that low-ratio LA/ALA supplementation significantly reduces lipid levels, especially in North Americans, dyslipidemia, and type 2 diabetes, among others. Daily intake of more than 20 mL of ALA-rich flaxseed and canola oil or more than 20 g of ALA-rich nuts such as walnuts may be beneficial for blood lipids. Additional research is warranted, encompassing diverse geographical locations and ethnicities, to evaluate the long-term effects of the inclusion of more rigorous RCTs.

## Figures and Tables

**Table 1:** Characteristics of the included studies.

Reference	Country	Participants Information	Age	BMI	Smoking	No.	M/F	Duration	Design	Low LA/ALA	High LA/ALA
Akrami 2018 [48]	Iran	Metabolic syndrome	48.6	NR	Non-smoker	52	33/19	7 W	P	0.14	19.1
Baxheinrich 2012 [49]	Germany	Metabolic syndrome	51.3	34.3	Non-smoker	81	26/55	24 W	P	3.12	10.89
Bemelmans 2002 [50]	Netherlands	Cardiovascular	54.1	29.8	Mixed	163	79/84	104 W	P	4.7	29.1
Chen 2020 [32]	China	Dyslipidaemia	54.5	23.2	Mixed	243	92/151	48 W	P	7.1	30
										7.1	20
Clandinin 1997 [20]	Canada	Healthy	30.9		Non-smoker	26	NR	12 W	CO	0.25	14.45
Damsgaard 2008 [21]	Denmark	Healthy	25	23.2	Mixed	33	33/0	8 W	P	4.7	7.72
Dittrich 2015 [33]	Germany	Dyslipidaemia	56	28.2	NR	49	17/32	10 W	CO	0.42	71.43
										0.74	71.43
Dobrzynska 2021 [34]	Poland	Dyslipidaemia	56.0	26.7	Mixed	60	0/60	6 W	P	0.48	1.38
Finnegan 2003 [35]	UK	Dyslipidaemia	53.7	26.1	Non-smoker	60	35/25	24 W	P	3.6	15.5
			54.5	26.2		59	35/24			1.4	15.5
Gillingham 2011 [15]	Canada	Dyslipidaemia	47.5	28.6	Non-smoker	39	14/25	4 W	CO	0.66	11.8
										0.66	6
Goyens 2005 [22]	Netherlands	Healthy	49.6	24.1	Mixed	36	14/22	6 W	P	7	19
Griffin 2006 [23]	UK	Healthy	59	26.3	Mixed	97	62/35	24 W	P	4.64	14
Jones 2014 [46]	Canada	Overweight or obese	46.5	29.8	Non-smoker	130	60/70	4 W	CO	1.17	231
Kaul 2008 [24]	Canada	Healthy	33.8	24.3	Non-smoker	44	17/27	12 W	P	0.29	45.8
										3.22	45.8
Kawakami 2015 [25]	Japan	Healthy	44.5	25.1	Mixed	15	15/0	12 W	CO	1.34	9.8
Kontogianni 2013 [26]	Greece	Healthy	26	21.9	NR	37	8/29	6 W	CO	1.4	8.3
Lee 2014 [42]	USA	T2DM	58.6	34.5	Non-smoker	43	18/25	8 W	P	0.95	66
Lichtenstein 2006 [36]	USA	Dyslipidaemia	64	25.7	Non-smoker	14	14/0	7 W	CO	8.7	18.3
			61	26.7		16	0/16			8.7	18.3
Ma 2010 [43]	USA	T2DM	58.1	32.5	Non-smoker	24	10/14	8 W	P	4.48	7.75
Mantzioris 1994 [27]	Australia	Healthy	35.3	25.4	NR	30	30/0	4 W	P	0.62	19.5
McManus 1996 [44]	Canada	T2DM	61.8	27.8	NR	11	8/3	12 W	CO	0.25	14.45
Minihane 2005 [28]	India	Healthy	48	26	Non-smoker	29	NR	6 W	P	9	16
Moszak 2020 [47]	Poland	Overweight or obese	48.7	39.6	Non-smoker	52	20/32	3 W	P	1.88	41.5
Pang 1998 [29]	Australia	Healthy	24.5	22.4	NR	29	29/0	6 W	P	0.89	67
Paschos 2007 [37]	Greece	Dyslipidaemia	52	28	Non-smoker	35	35/0	12 W	P	0.26	148
Rallidis 2003 [38]	Greece	Dyslipidaemia	51	28.4	Mixed	76	76/0	12 W	P	1.3	13.2
Rezaei 2020 [51]	Iran	Non-alcoholic fatty liver	43.2	29.9	Mixed	68	33/35	12 W	P	0.36	228.2
Schwab 2006 [30]	USA	Healthy	45	24.5	NR	14	8/6	4 W	CO	0.245	2.45
Schwab 2018 [45]	Finland	T2DM	58.9	29.2	NR	79	40/39	12 W	P	1.1	4.3
Sodergren 2001 [39]	Sweden	Dyslipidaemia	50	24.5	Mixed	19	13/6	4 W	CO	3	10
Wilkinson 2005 [31]	UK	Healthy	49	28.3	Non-smoker	38	NR	12 W	P	0.5	27.9
Zhao 2005 [40]	USA	Dyslipidaemia	49.8	28.1	Non-smoker	23	20/3	6 W	CO	1.5	9
										3.5	9
Zhou 2019 [41]	China	Dyslipidaemia	52.7	26	Mixed	75	39/36	12 W	P	2.05	16.04

Abbreviations: T2DM, type 2 diabetes; BMI, body mass index; NR, not reported; No., number of included participants; M, male; F, female; W, weeks; P, parallel; CO, crossover; LA/ALA, linoleic acid/alpha-linolenic acid.

**Table 2:** Quality assessment of the included studies.

Study	Random Sequence Generation	Allocation Concealment	Blinding of Participants and Personnel	Blinding of Outcome Assessments	Incomplete Outcome Data	Selective Outcome Reporting	Other Bias
Akrami 2018	L	U	U	U	L	L	L
Baxheinrich 2012	U	U	U	U	L	L	L
Bemelmans 2002	L	L	L	U	L	U	L
Chen 2020	L	L	L	U	L	U	L
Clandinin 1997	L	U	L	L	L	U	L
Damsgaard 2008	L	L	L	L	L	L	L
Dittrich 2015	U	U	L	U	L	U	L
Dobrzynska 2021	U	L	L	U	L	U	L
Finnegan 2003	L	U	L	U	L	U	L
Gillingham 2011	L	L	H	U	L	L	L
Goyens 2005	U	U	L	U	L	U	L
Griffin 2006	U	U	U	U	U	U	L
Jones 2014	L	U	L	U	L	L	L
Kaul 2008	L	U	L	U	U	U	L
Kawakami 2015	U	U	L	U	L	U	L
Kontogianni 2013	L	L	H	U	L	U	L
Lee 2014	U	U	H	U	L	U	L
Lichtenstein 2006	U	U	U	U	L	U	L
Ma 2010	U	U	L	L	L	L	L
Mantzioris 1994	U	U	U	U	L	U	L
McManus 1996	L	U	L	L	L	U	L
Minihane 2005	U	U	L	U	L	U	L
Moszak 2020	U	U	L	L	L	U	L
Pang 1998	U	U	U	U	U	U	L
Paschos 2007	U	U	H	U	U	U	L
Rallidis 2003	U	U	U	U	U	U	L
Rezaei 2020	L	L	L	U	L	L	L
Schwab 2006	U	U	L	U	U	U	L
Schwab 2018	L	U	U	L	L	U	L
Sodergren 2001	U	U	U	U	U	U	L
Wilkinson 2005	U	U	H	U	U	U	L
Zhao 2005	L	U	U	L	L	U	L
Zhou 2019	U	U	L	U	L	L	L

H: high risk of bias; U: unclear risk of bias; L: low risk of bias.

**Table 3:** Subgroup analysis of low-ratio LA/ALA on TG, TC, HDL-C, and LDL-C.

		TG			TC			HDL			LDL	
Subgroup	N	WMD (95% CI)	I^2^ %	N	WMD (95% CI)	I^2^ %	N	WMD (95% CI)	I^2^ %	N	WMD (95% CI)	I^2^ %
Low-ratio LA/ALA												
≤1	16	−0.04 (−0.13, 0.05)	0.00	16	−0.03 (−0.19, 0.13)	32.6	16	−0.01 (−0.05, 0.02)	0.0	15	0.01 (−0.11, 0.12)	0.0
1–5	18	−0.04 (−0.10, 0.02)	1.2	18	−0.12 (−0.23, −0.01)	34.6	18	0.00 (−0.03, 0.02)	0.0	18	−0.10 (−0.18, −0.02)	8.3
≥5	6	0.05 (−0.21, 0.30)	46.7	6	−0.08 (−0.28, 0.11)	45.4	6	0.04 (−0.02, 0.11)	0.0	6	−0.11 (−0.25, 0.04)	5.4
Region												
North America	13	−0.06 (−0.14, 0.02)	0.0	13	−0.21 (−0.33, −0.07)	22.7	13	−0.01 (−0.04, 0.02)	0.0	13	−0.14 (−0.23, −0.05)	7.7
Europe	17	−0.03 (−0.11, 0.05)	11.8	17	−0.06 (−0.22, 0.09)	49.2	17	0.00 (−0.04, 0.03)	0.0	16	−0.07 (−0.16, 0.03)	0.0
Asia	8	0.05 (−0.11, 0.20)	0.0	8	0.02 (−0.12, 0.15)	0.0	8	0.01 (−0.03, 0.05)	0.0	8	0.01 (−0.13, 0.14)	0.0
Oceania	2	−0.12 (−0.27, 0.03)	0.0	2	0.15 (−0.15, 0.45)	0.0	2	0.05 (−0.08, 0.17)	0.0	2	0.19 (−0.09, 0.46)	0.0
Health status												
Health	13	−0.08 (−0.15, −0.01)	0.0	13	−0.15 (−0.28, −0.03)	8.0	13	−0.01 (−0.05, 0.04)	0.0	13	−0.09 (−0.19, 0.02)	0.0
Dyslipidaemia	17	0.04 (−0.07, 0.14)	3.3	17	−0.08 (−0.24, 0.09)	55.4	17	0.01 (−0.03, 0.04)	0.0	16	−0.14 (−0.25, −0.03)	5.9
T2DM	4	−0.07 (−0.18, 0.04)	0.0	4	−0.12 (−0.24, 0.01)	0.0	4	0.01 (−0.03, 0.05)	0.0	4	−0.13 (−0.24, −0.01)	0.0
Overweight or obese	2	−0.05 (−0.27, 0.16)	0.0	2	−0.12 (−0.40, 0.16)	0.0	2	−0.02 (−0.09, 0.04)	0.0	2	0.00 (−0.21, 0.20)	0.0
Other	4	0.00 (−0.19, 0.19)	0.0	4	0.11 (−0.05, 0.27)	0.0	4	−0.01 (−0.05, 0.03)	0.0	4	0.11 (−0.03, 0.25)	0.0
Age												
≤45	20	−0.09 (−0.15, −0.03)	0.0	20	−0.20 (−0.32, −0.09)	32.3	20	−0.01 (−0.04, 0.01)	0.0	20	−0.11 (−0.21, 0.02)	25.0
>45	20	0.01 (−0.06, 0.08)	0.0	20	0.00 (−0.08, 0.08)	0.0	20	0.01 (−0.02, 0.04)	0.0	19	−0.05 (−0.12, 0.03)	0.0
BMI												
≤25	7	−0.09 (−0.20, 0.02)	34.0	7	−0.10 (−0.28, 0.08)	52.3	7	0.04 (−0.02, 0.09)	0.3	7	−0.01 (−0.26, 0.07)	40.6
25–30	22	0.02 (−0.07, 0.10)	0.0	22	−0.10 (−0.24, 0.04)	42.1	22	−0.02 (−0.05, 0.02)	0.0	21	−0.11 (−0.20, −0.02)	0.0
≥30	8	0.08 (−0.18, 0.02)	0.0	8	−0.12 (−0.23, 0.00)	0.0	8	0.01 (−0.03, 0.05)	5.1	8	−0.11 (−0.21, −0.01)	0.0
NR	3	0.06 (−0.09, 0.21)	0.0	3	0.13 (−0.05, 0.31)	0.0	3	−0.02 (−0.06, 0.02)	0.0	3	0.14 (−0.01, 0.30)	0.0
Smoking												
Non-smoker	20	−0.03 (−0.10, 0.04)	0.0	20	−0.12 (−0.25, 0.01)	45.9	20	−0.00 (−0.03, 0.02)	0.0	19	−0.09 (−0.17, −0.02)	5.6
Mixed	13	−0.01 (−0.11, 0.09)	20.7	13	−0.05 (−0.16, 0.07)	22.2	13	0.00 (−0.04, 0.04)	13.2	13	−0.06 (−0.15, 0.04)	0.2
NR	7	−0.09 (−0.19, 0.01)	0.0	7	−0.08 (−0.28, 0.13)	7.5	7	0.01 (−0.06, 0.08)	0.0	7	−0.03 (−0.20, 0.15)	2.3
Duration												
<12 W	22	−0.09 (−0.15, −0.04)	0.0	22	−0.18 (−0.28, −0.08)	26.7	22	0.00 (−0.02, 0.02)	0.0	22	−0.13 (−0.21, −0.04)	21.8
≥12 W	18	0.06 (−0.02, 0.15)	0.0	18	0.03 (−0.07, 0.14)	9.4	18	0.00 (−0.03, 0.03)	0.0	17	0.02 (−0.08, 0.11)	0.0

Abbreviations: CI, confidential interval; N, number of included studies; LA/ALA, linoleic acid/alpha-linolenic acid; T2DM, type 2 diabetes; BMI, body mass index; NR, not reported; W, weeks.

## Data Availability

Data is contained within the article and Supplementary Material.

## Supplementary Materials

The following supporting information can be downloaded at: https://www.mdpi.com/article/10.3390/foods12163005/s1, Figure S1: Funnel plots of low-ratio LA/ALA and TG (A), TC (B), HDL-C (C) and LDL-C (D). Figure S2: Sensitivity analysis of low-ratio LA/ALA and TG. Figure S3: Sensitivity analysis of low-ratio LA/ALA and TC. Figure S4: Sensitivity analysis of low-ratio LA/ALA and HDL-C. Figure S5: Sensitivity analysis of low-ratio LA/ALA and LDL-C. Table S1: Dietary intakes of nutrients of the participants during the intervention period.

## Abbreviations

LA	Linoleic acid
ALA	Alpha-linolenic acid
RCTs	Randomized controlled trials
WMD	Weighted mean difference
CI	Confidence interval
TG	Triglycerides
TC	Total cholesterol
HDL-C	High-density lipoprotein cholesterol
LDL-C	Low-density lipoprotein cholesterol
CVD	Cardiovascular disease
PUFA	Polyunsaturated fatty acid
EPA	Eicosapentaenoic acid
DHA	Docosahexaenoic acid
CLA	Conjugated linoleic acid
BMI	Body mass index
SD	Standard deviations
MCFA	Medium-chain fatty acids
PPAR	Peroxisome proliferator-activated receptors
NF-κB	Nuclear factor-kappa B
