# Supplementary material for: Effects of Dietary Plant-Derived Low-Ratio Linoleic Acid/Alpha-Linolenic Acid on Blood Lipid Profiles: A Systematic Review and Meta-Analysis
Source: Foods. 2023 Aug 9;12(16):3005. doi: 10.3390/foods12163005 (PMC10453764; doi:10.3390/foods12163005)
Supplement: Supplementary file 1 [file foods-12-03005-s001.zip › Figure.pdf]

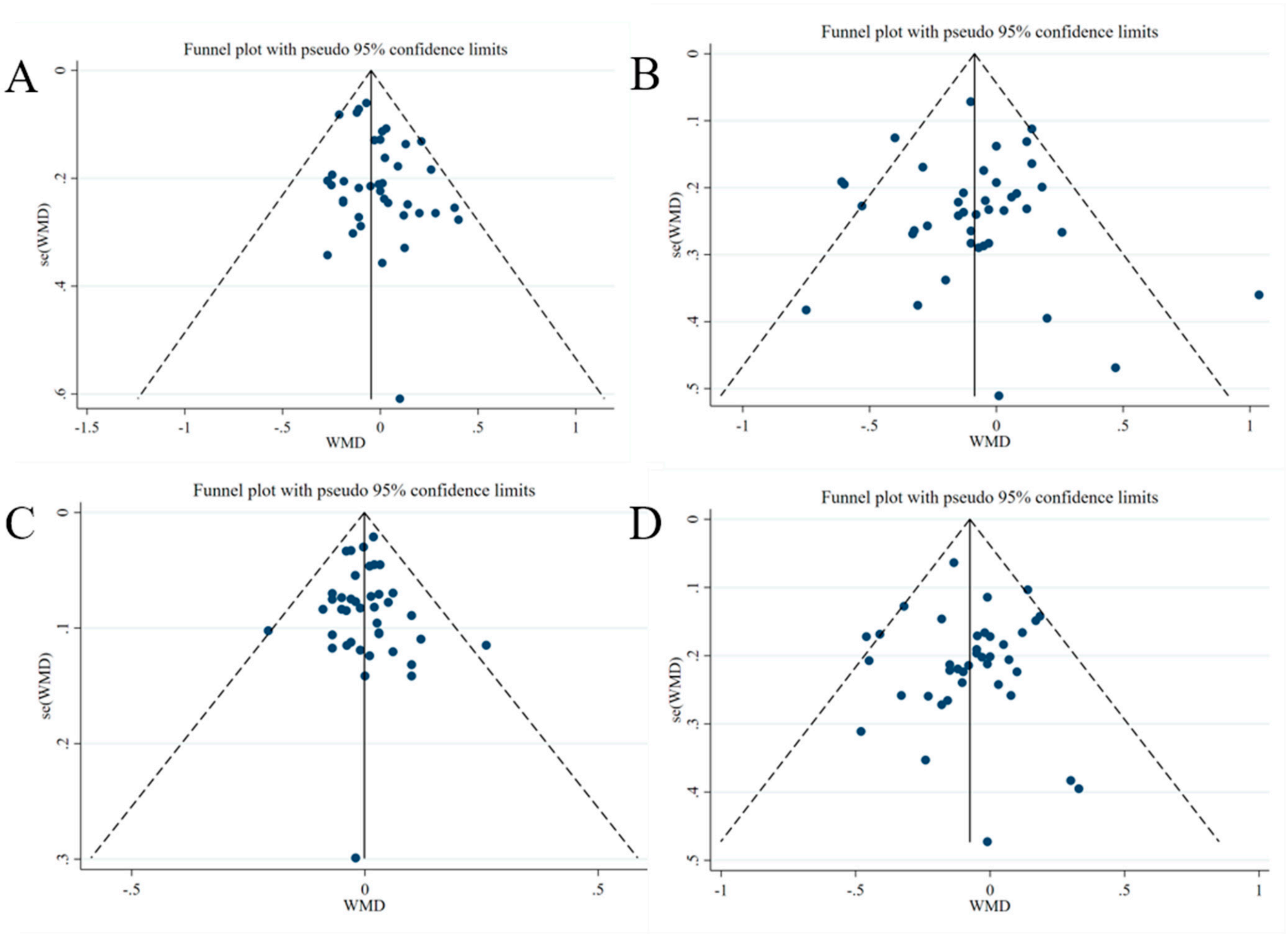

Figure S1 Funnel plots of low-ratio LA/ALA and TG (A), TC (B), HDL-C (C) and LDL-C (D).

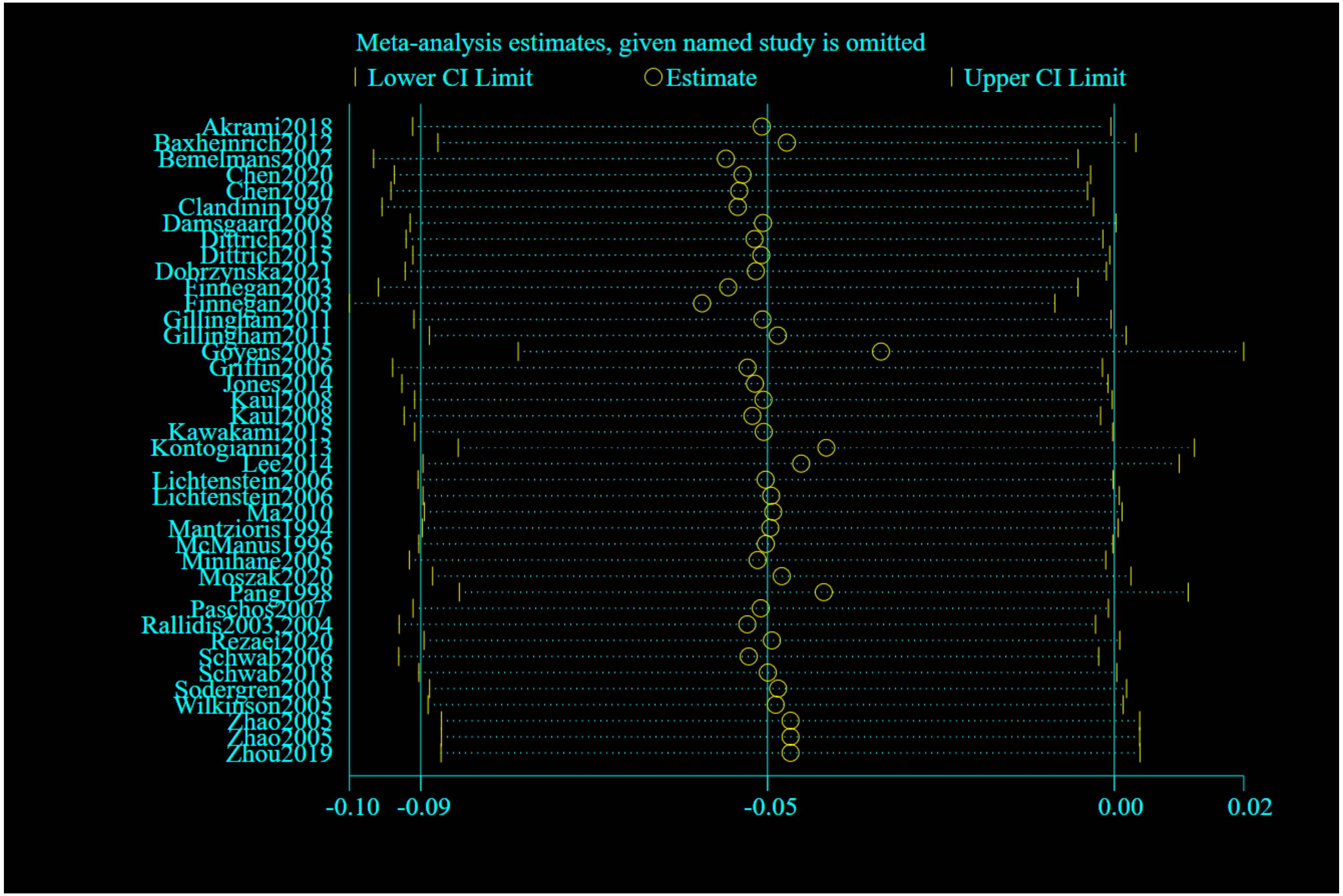

Figure S2 Sensitivity analysis of low-ratio LA/ALA and TG.

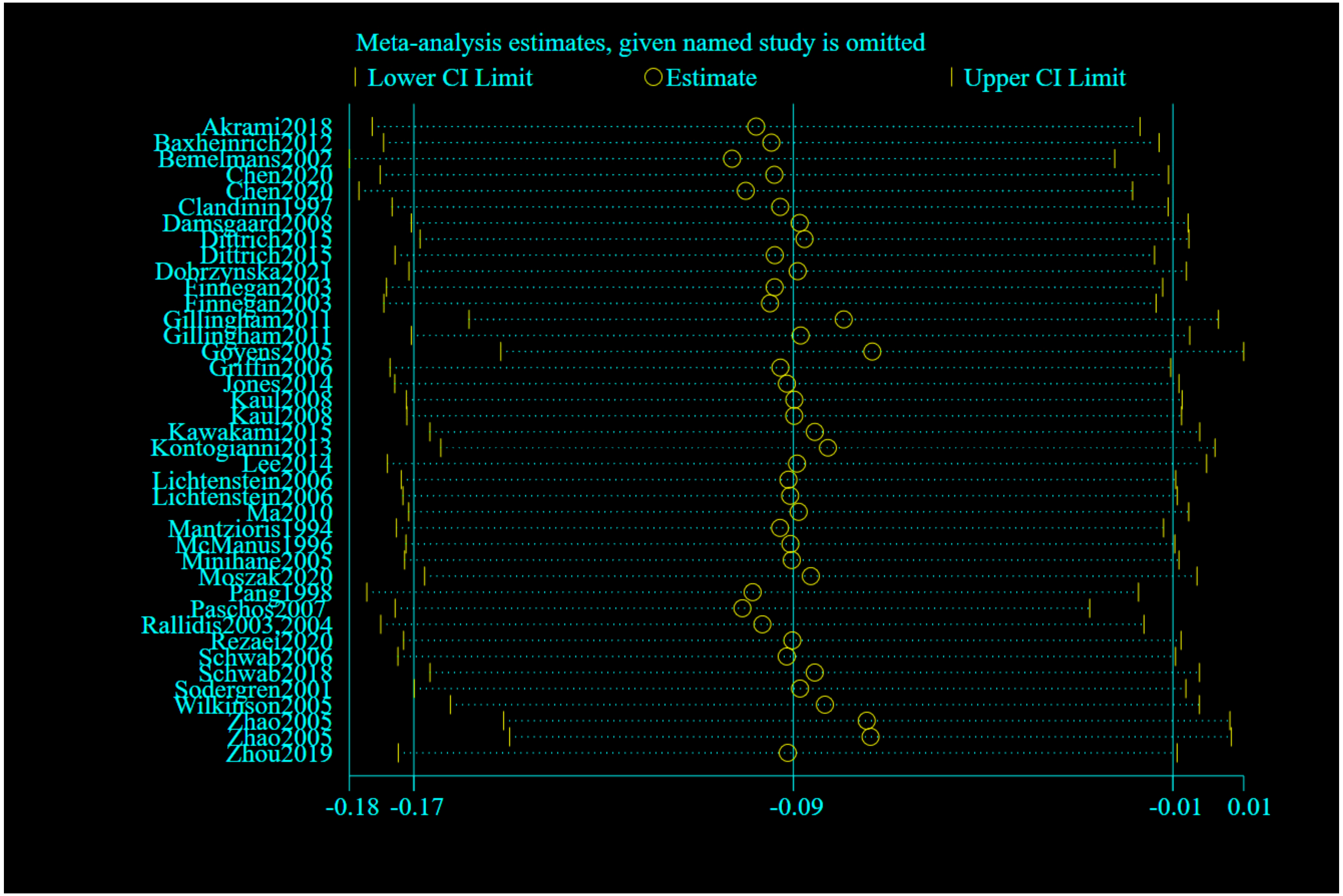

Figure S3 Sensitivity analysis of low-ratio LA/ALA and TC.

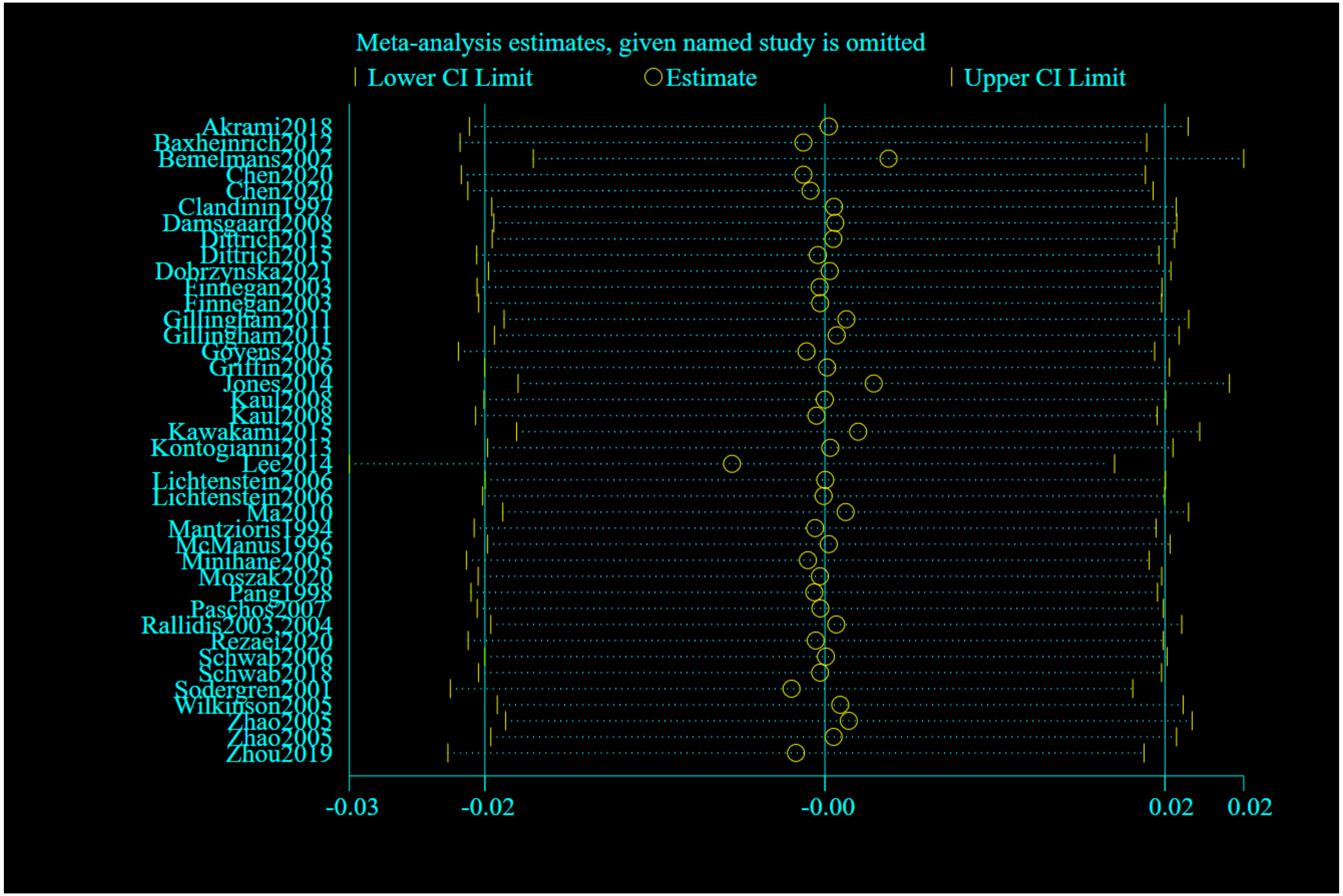

Figure S4 Sensitivity analysis of low-ratio LA/ALA and HDL-C.

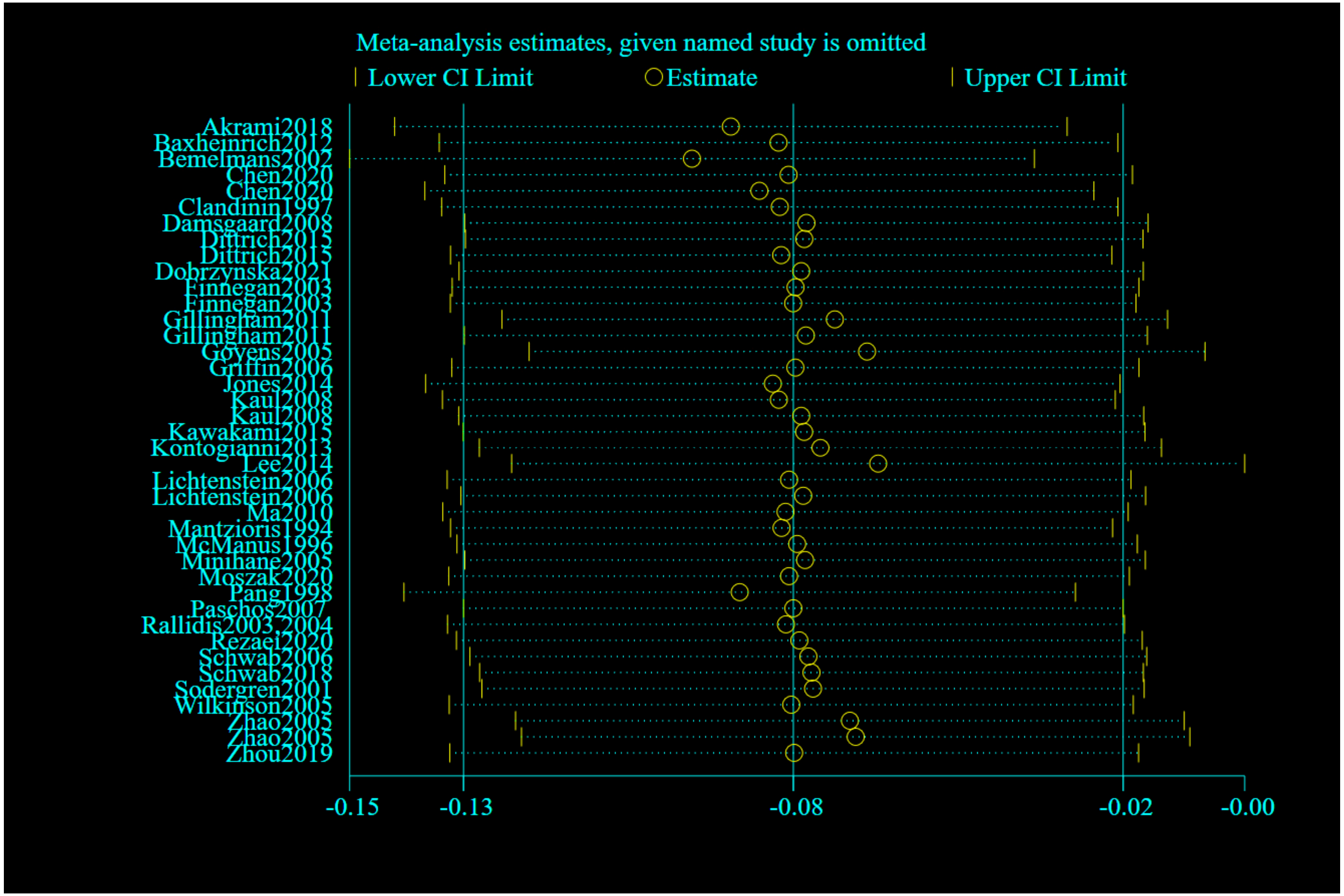

Figure S5 Sensitivity analysis of low-ratio LA/ALA and LDL-C.
