# Supplementary material for: Effects of Dietary Plant-Derived Low-Ratio Linoleic Acid/Alpha-Linolenic Acid on Blood Lipid Profiles: A Systematic Review and Meta-Analysis
Source: Foods. 2023 Aug 9;12(16):3005. doi: 10.3390/foods12163005 (PMC10453764; doi:10.3390/foods12163005)
Supplement: Supplementary file 1 [file foods-12-03005-s001.zip › Table S1.pdf]

Table S1 Dietary intakes of nutrient of the participants at intervention period

Reference	Group	Energy (kcal/d)	Protein	Carbohydrate	Fat	SFA	MUFA	PUFA	Total LA	Total ALA	Supplementation LA (g/d)	Supplementation ALA (g/d)	LA and ALA Source
			(% of total energy intake/d)										
Akrami2018	Low LA/ALA	2481	15.1	56.3	28.4	7.5	3.6	17.3[b]	2.1[a]	15.2[b]			25 ml flaxseed oil
	High LA/ALA	2490	14.8	56.2	28.8	7.6	4.7	16.5[a]	15.68[b]	0.82[a]			25 ml sunflower oil
Baxheinrich2012	Low LA/ALA	1649.1	19.9	40.4	38.5	9.8	18.3	8.1[b]	10.8 g	3.46[b] g			30 g rapeseed oil plus 20 g margarine
	High LA/ALA	1577.4	18.7	42.4	38.5	10.8	19.6	5.5[a]	8.5 g	0.78[a] g			30 g olive oil plus 20 g margarine
Bemelmans2002	Low LA/ALA	2796.3			37.7	13.5	12.1	12.1	9.5	2.3[b]			ALA-rich margarine
	High LA/ALA	2796.3			37.7	13.5	12.1	12.1	10.6	0.4[a]			LA-rich margarine
Chen2020	Low LA/ALA	2248.2	16.3	52.0	31.7	7.2	11.9	7.5	7.1	1.0[b]			27 g blend oil
	High LA/ALA	2161.4	16.1	53.4	30.5	7.4	11.3	6.8	6.3	0.21[a]			27 g peanut oil
	High LA/ALA	2072.3	15.5	54.6	29.8	7.2	9.4	8.4	7.8	0.39[a]			27 g corn oil
Clandinin1997	Low LA/ALA	1910	14.5	51.3	32.6	11.5					14.2% of total fatty caids	57.5% of total fatty caids	35 mg/kg body weight flaxseed oil
	High LA/ALA	1910	14.5	51.3	32.6	11.5					13% of total fatty caids	0.9% of total fatty caids	35 mg/kg body weight olive oil
Damsgaard2008	Low LA/ALA	2722	15	48	36	12.2	13.2	5[a]	12.3[a] g	2.6 g			10.3 g (0.0-52.5g/d) rapeseed oil
	High LA/ALA	2654.6	14	51	35	10.5	11.5	7.5[b]	19.3[b] g	2.5 g			10.3 g (0.0-52.5g/d) sunflower oil

Table S1. Cont.

Reference	Group	Energy (kcal/d)	Protein	Carbohydrate	Fat	SFA	MUFA	PUFA	Total LA	Total ALA	Supplementation LA (g/d)	Supplementation ALA (g/d)	LA and ALA Source
			(% of total energy intake/d)										
Dittrich2015	Low LA/ALA	1871.01	15.95	39.93	38.62	16.77	14.01	4.81	3.1[a] g	7.42[c] g			20 g linseed oil
	Low LA/ALA	2182.58	16.30	42.26	35.12	14.30	12.93	5.43	3.57[a] g	4.81[b] g			20 g echium oil
	High LA/ALA	1873.06	16.18	42.75	36.6	15.56	12.86	5.29	10.0[b] g	0.14[a] g			20 g sunflower oil
Dobrzynska2021	Low LA/ALA	2144	15.3	49.3	39.0	13.2	15.4	6.9[a]			5.07 g	10.59[b] g	30 g camelina oil
	High LA/ALA	2170	15.5	49.0	39.1	11.9	15.5	8.3[b]			5.25 g	3.81[a] g	30 g canola oil
Finnegan2003	Low LA/ALA	2342.2	15.5	45.3	36.0	11.5	10.1	8.4	16.2[a] g	4.5[b] g			25 g ALA margarine (rapeseed and linseed oils)
	Low LA/ALA	2485.6	15.4	43.7	37.8	12.3	10.9	8.8	13.1[a] g	9.5[c] g			25 g ALA margarine (rapeseed and linseed oils)
	High LA/ALA	2891.9	16.1	43.3	36.2	11.9	10.5	8.2	22.9[b] g	1.5[a] g			25 g LA margarine (sunflower and safflower oils)
Gillingham2011	Low LA/ALA	2500	14.4	48.7	36.9	6.1[a]	15.9[a]	12.3[b]	4.9	7.4[b]			Flaxseed and high oleic rapeseed oil diet
	High LA/ALA	2500	14.4	48.8	36.8	11.2[b]	16.1[a]	6.5[a]	5.9	0.5[a]			Western diet（SFA）
	High LA/ALA	2500	14.4	48.8	36.8	5.6[a]	22.9[b]	5.7[a]	4.8	0.8[a]			High oleic rapeseed oil

Table S1. Cont.

Reference	Group	Energy (kcal/d)	Protein	Carbohydrate	Fat	SFA	MUFA	PUFA	Total LA	Total ALA	Supplementation LA (g/d)	Supplementation ALA (g/d)	LA and ALA Source
			(% of total energy intake/d)										
Goyens2005	Low LA/ALA	2581.2	15.5	50.4	32.6	10.4	12.6	8.6	7.1	1.1[b]			52.5% rapeseed oil, 20% sunflower oil, 5% olive oil, 22.5% fully hydrogenated palm kernel and palm oil
	High LA/ALA	2509.5	14.5	50.5	33.5	11.6	12.8	8.0	7.3	0.4[a]			30.4% sunflower oil, 33.1% olive oil, 11.5% rapeseed oil, 25% fully hydrogenated palm kernel and palm oil
Griffin2006	Low LA/ALA	2093.6	16.4	42.3	36.1	11.5	14.2	6.8[a]	5.1[a]	1.1[b]			32 g rapeseed oil
	High LA/ALA	2148.6	16.4	41.0	37.4	12.2	14.4	8.2[b]	7[b]	0.5[a]			32 g high-oleic sunflower oil
Jones2014	Low LA/ALA	3000	15	50	35	6.7	9.5	16.3	8.79[a]	7.51[b]			60 g flaxseed and safflower oil
	High LA/ALA	3000	15	50	35	6.8	9.6	16.3	16.22[b]	0.07[a]			60 g corn and safflower oil
Kaul2008	Low LA/ALA										0.298[a]	1.022[b]	2 g flaxseed oil
	Low LA/ALA										1.196[b]	0.372[a]	2 g hempseed oil
	High LA/ALA										1.374[b]	0.3[a]	2 g sunflower oil

Table S1. Cont.

Reference	Group	Energy (kcal/d)	Protein	Carbohydrate	Fat	SFA	MUFA	PUFA	Total LA	Total ALA	Supplementation LA (g/d)	Supplementation ALA (g/d)	LA and ALA Source
			(% of total energy intake/d)										
Kawakami2015	Low LA/ALA	2261.4	13.02	55.75	25.51				8.11[a]	6.03[b]			10 g flaxseed oil
	High LA/ALA	2289.8	13.33	53.51	28.42				15.68[b]	1.6[a]			10 g corn oil
Kontogianni2013	Low LA/ALA	1929.2	15.9	44.2	37.0	12.3	14.5[a]	8.4[b]		8.7[b] g			15 ml flaxseed oil
	High LA/ALA	1806.1	18.4	47.6	39.0	12.0	17.6[b]	6.0[a]		0.8[a] g			15 ml olive oil
Lee2014	Low LA/ALA										1.8[a]	1.9[b]	7 echium oil capsules and 3 borage oil capsules
	High LA/ALA										3.96[b]	0.06[a]	9 corn oil capsules
Lichtenstein2006	Low LA/ALA	2521	16.7	52.1	31.2	6.52	6.48	12.74	10.96[a]	1.26[b]			Soybean oils provide 20% of total energy
	High LA/ALA	2521	16.8	52.1	31.1	6.75	6.96	13.51	12.45[b]	0.68[a]			Low-α-linolenic acid soybean oil provide 20% of total energy
Ma2010	Low LA/ALA	1765	17	39	45[b]	10.88	9.18	16.83[b]	26.0[b] g	5.8[b] g			56 g of shelled, unroasted English walnuts
	High LA/ALA	1685	19	43	38[a]	12.52	9.61	4.91[a]	6.2[a] g	0.8[a] g			Ad libitum diet without walnuts

Table S1. Cont.

Reference	Group	Energy (kcal/d)	Protein	Carbohydrate	Fat	SFA	MUFA	PUFA	Total LA	Total ALA	Supplementation LA (g/d)	Supplementation ALA (g/d)	LA and ALA Source
			(% of total energy intake/d)										
Mantzioris1994	Low LA/ALA	2198.8	18.9	47.2	29.6				3.3[a]	5.3[b]			Flaxseed oil
	High LA/ALA	2294.4	19.0	47.9	29.1				7.8[b]	0.4[a]			High n-6 fatty acids cooking oils
McManus1996	Low LA/ALA										14.2% of total fatty caids	57.5% of total fatty caids	35 mg/kg body weight flaxseed oil
	High LA/ALA										13% of total fatty caids	0.9% of total fatty caids	35 mg/kg body weight olive oil
Minihane2005	Low LA/ALA				39	9	15[b]	5.7[a]	5[a]	0.7			olive oil
	High LA/ALA				37	10	10[a]	10.7[b]	10[b]	0.7			coin oil
Moszak2020	Low LA/ALA		20%	50-55%	25-30%						4.2[a]	2.24[b]	20 ml rapeseed oil
	High LA/ALA		20%	50-55%	25-30%						9.98[b]	0.24[a]	20 ml amaranth seed oil
Pang1998	Low LA/ALA	3144.5	15.5	49.1	31.2	9.7	11.6	6.7	3.1[a]	3.5[b]			Linseed oil
	High LA/ALA	3092.9	15.5	49.1	31.4	9.8	11.8	6.9	6.7[b]	0.1[a]			Safflower oil
Paschos2007	Low LA/ALA	2189	15.3	47.7	37.1	13.44	16.73	5.10			2.09[a]	8.1[b]	15 ml flaxseed oil
	High LA/ALA	2208	15.2	48	36.8	12.55	16.79	5.58			11.2[b]	0.08[a]	15 ml safflower oil
Rallidis2003	Low LA/ALA	2181.8	15.1	47.7	35.9	9.41	19.18	4.91	10.6 g	8[b] g			15 ml linseed oil
	High LA/ALA	2183.2	14.9	47.8	35.9	9.56	19.21	4.95	11 g	0.83[a] g			15 ml safflower oil
Rezaei2020	Low LA/ALA	1807	66.30	13.17	20.77	6.18	5.58	8.82			3.62[a]	9.96[b]	20 g flaxseed oil
	High LA/ALA	1781	63.83	13.32	22.99	6.52	6.77	9.75			12.68[b]	0.044[a]	20 g sunflower oil

Table S1. Cont.

Reference	Group	Energy (kcal/d)	Protein	Carbohydrate	Fat	SFA	MUFA	PUFA	Total LA	Total ALA	Supplementation LA (g/d)	Supplementation ALA (g/d)	LA and ALA Source
			(% of total energy intake/d)										
Schwab2006	Low LA/ALA	2044.4	15.5	42.8	33.8	13.2	11.4	5.3			3.9[a]	15.9[b]	30 ml flaxseed oil
	High LA/ALA	2044.6	15.7	43.6	32.4	12.1	11.1	5.3			16.2[b]	6.6[a]	30 ml hemp seed oil
Schwab2018	Low LA/ALA	2215.5	15.6[a]	38.2[a]	42.5[b]	12.1	15.0	11.6[b]	13.5[b] g	12.4[b] g			27 g camelina sativa oil and canola oil
	High LA/ALA	1938.5	17.7[b]	42.5[b]	34.0[a]	11.3	13.1	5.6[a]	9.0[a] g	2.1[a] g			27 g canola oil and olive oil
Sodergren2001	Low LA/ALA	2127.1	14.8	47.3	35.9	10.3[a]	15.7[b]	7.5[b]	5.4[b]	1.8[b]			Rapeseed oil provide 27% of total energy
	High LA/ALA	2318.3	14.4	47.5	36.2	17.6[b]	10.9[a]	4.6[a]	4.0[a]	0.4[a]			Butter and olive oil provide 27% of total energy
Wilkinson2005	Low LA/ALA	2603	15	39	41	11.8	17.8[b]	9.5[a]	8.7[a] g	19[b] g			45 g Flaxseed oil
	High LA/ALA	2426	15	41	41	11.3	12.8[a]	11.1[b]	25.2[b] g	1.5[a] g			45 g Sunflower oil
Zhao2005	Low LA/ALA	2397.6	16.1	46.3	37.6[b]	8.2[a]	12.3	17.2b	10.5[b]	6.5[c]			Flaxseed oil, walnuts, and walnut oil
	Low LA/ALA	2397.6	16.1	46.8	37.1[b]	8.5[a]	12.2	16.4b	12.6[c]	3.6[b]			Walnuts and walnut oil
	High LA/ALA	2397.6	15.7	49.8	34.5[a]	12.7[b]	13.2	8.7a	7.7[a]	0.8[a]			Average American diet

Table S1. Cont.

Reference	Group	Energy (kcal/d)	Protein	Carbohydrate	Fat	SFA	MUFA	PUFA	Total LA	Total ALA	Supplementation LA (g/d)	Supplementation ALA (g/d)	LA and ALA Source
			(% of total energy intake/d)										
Zhou2019	Low LA/ALA	1927			33.72	7.89	10.13	11.44	16.8[a] g	8.18[b] g			25 ml flaxseed oil and corn oil
	High LA/ALA	1960			34.35	8.17	11.53	10.70	21.5[b] g	1.34[a] g			25 ml corn oil

SFA, saturated fatty acids; MUFA, monounsaturated fatty acids; PUFA, polyunsaturated fatty acids; LA, linoleic acid; ALA, α-linolenic acid; Different letters indicate significant differences ($p < 0.05$) between each group, and the same letters indicate that there is no significant difference ($p > 0.05$) between each group.
